# Assessing the recovery of steroid levels and gonadal histopathology of tilapia exposed to polystyrene particle pollution by supplementary feed

**DOI:** 10.14202/vetworld.2022.517-523

**Published:** 2022-02-28

**Authors:** Alfiah Hayati, Manikya Pramudya, Hari Soepriandono, Aisyah Rizkyning Astri, Michael Ronaldi Kusuma, Sasanaqia Maulidah, Wahyu Adriansyah, Firli Rahmah Primula Dewi

**Affiliations:** Department of Biology, Faculty of Science and Technology, Universitas Airlangga, Campus C, Mulyorejo, Surabaya, Indonesia

**Keywords:** fish, fisheries, freshwater, polystyrene, probiotics, testicular

## Abstract

**Background and Aim:**

Water pollution caused by industrial waste and human activities has disrupted the reproductive health of aquatic organisms. This study aimed to analyze the effects of water pollution caused by polystyrene particles (PP) on the steroid (estradiol and testosterone) levels and histopathology of male tilapia gonads. In addition, we also analyzed the potential of supplementary feeding to remove and neutralize oxidants.

**Materials and Methods:**

Thirty-six tilapia fishes were taken for the study and were divided into 12 groups (n=3), including a control group (fed with commercial pellets only) and groups fed with a mixture of commercial-probiotic pellets (200 mL/kg, 1×10^8^ colony-forming unit [CFU]/mL) and commercial vitamin C pellets (100 mg/kg), respectively. The PP concentrations used for this study were 0, 0.1, 1, and 10 mg/L, and the treatment time was 2 weeks. The testosterone and estradiol concentrations were analyzed by enzyme-linked immunosorbent assay and histopathological analysis of the gonads.

**Results:**

Laboratory analysis performed using tilapia fishes showed that exposure to a PP concentration of <74 μm, mixed with feed for 14 days, could decrease estradiol and testosterone levels. Exposure to plastic particles could change the structure, shape, and size of male gonads. It can also affect the spermatogenic cell number and alter the diameter inside the cysts. Originally, plastic particles were believed to reduce the permeability of the cyst membrane, and this damages the membrane or ruptures the cyst. Supplementary feed containing probiotics (200 mL/kg, 1×108 CFU/mL) and vitamin C (100 mg/kg) can ameliorate the impact of PP exposure on steroid levels. The steroid levels increase with a concurrent improvement in cysts and seminiferous tubule structures.

**Conclusion:**

Overall, this study indicates that PP concentrations in the aquatic environment negatively affect tilapia reproduction, and this may pose a potential threat to the fish population in freshwater. Provision of supplementary feed containing probiotics and vitamin C may serve as an alternative way to counter the negative impact caused by plastic particles.

## Introduction

Fertilization of tilapia occurs externally by the fusion of egg cells and spermatozoa outside the body. The success rate of fertilization in waters is determined by the physical and chemical conditions of the aquatic environment. However, it is also influenced by the quality of gamete cells (male gametes or spermatozoa) produced during spermatogenesis in the testes. Fish testes produce numerous small gametes, that is, spermatozoa. In tilapia, spermatogenic cells develop in cysts that make up the seminiferous tubules in the testes. The viability of spermatozoa in the fertility process highly depends on the development process and availability of steroid hormones. Many factors (heavy metals, plastic particles, etc.) can inhibit the process of spermatogenesis and the hormonal system of fish. These include toxic materials that enter through the gills and digestive tract after absorption through the blood capillaries and circulation throughout the body [1]. The concentration of toxic substances increases following the generation of reactive oxygen species, which damages cells and tissues, thereby resulting in cell death [2]. Plastic wastes are toxic materials that pollute water bodies [3]. The size classification of plastic waste includes large, macro, meso, and microdebris, which are of 100, 20, 20-5, and <5 mm, respectively. These plastics pollute water bodies, including seawater, rivers, and lakes [4,5].

Plastic polystyrene materials of larger sizes have been found in the digestive tract of fishes, disrupting the normal physiological function of the tract. Studies have shown that plastic waste fills the digestive tract and inhibits feed entry and absorption through the intestinal epithelium of fishes. Hence, fishes become starved for a long time, with reduced cellular function, and finally die. Large polystyrene particles (PP) are degraded over long periods in nature by ultraviolet light to form small particles called microplastics (size <0.1 μm-5 mm) and nanoplastics (particles <0.1 μm) [6]. These particles are directly or indirectly absorbed into the fish body through gills and the digestive tract. They can be circulated to all body tissues through blood capillaries. Plastic particles have been found in several organs of fish living in polluted areas, including the intestines, liver, gills [7], and brains [8]. At the cellular level, these toxic substances cause oxidative stress through the oxidation of lipids, proteins, and DNA molecules in the cell. This imbalance leads to inflammation and ultimately to cell death. Oxidative stress in the reproductive system results in infertility in the fish due to the dysfunction of the reproductive system. Several findings have shown that oxidative stress due to toxic substances in the fish body system reduces the function of the hypothalamic–pituitary–gonadal axis. This situation results in stunted growth and poor development of cells, which are conditions that depend on hormonal activity. Hence, the dysfunction of the hypothalamus due to toxic substances results in a reduction of gonadotropin-releasing hormone (GnRH) secretion. The GnRH level reduction is accompanied by a decreased synthesis of steroids, estradiol, and testosterone, thus affecting the development of gamete cells in the testes. Apart from producing gametes, testes are also considered endocrine organs that produce steroids. Oxidative stress also affects the structure and size of spermatogenic cells in the testes. It also damages the seminiferous tubule structure and inhibits the process of spermatogenesis, thus impairing the process of fertilization [9]. In nature, plastic particles can also bind to other materials, including other toxic materials such as heavy metals, copper, and zinc [4]. Heavy metals are also toxic materials from industrial waste, which are often discarded into rivers or the sea. Thus, the rivers and seawater are toxic due to uncontrolled human activities.

This study aimed to analyze the changes in the estradiol and testosterone levels and testicular histopathology of tilapia following exposure to PP. In addition, we seek to explore probiotic and vitamin C supplements as effective solutions to ameliorate and neutralize the toxic effects of PP in the digestive tract and reproductive system.

## Materials and Methods

### Ethical approval

All procedures involving treatment and experimentation on experimental fish were performed according to internationally recognized guidelines for the ethical use of animals [10-12]. Fish were quarantined and acclimatized before the experiment. Facilities (glass aquariums, water quality, and maintenance) are designed to minimize stress on reared fish [11]. Before being sacrificed, the fish were made unconscious in 0.1 mL/L clove oil containing eugenol. Clove oil solution was used to pacify the fish for several minutes without reacting to rejection in the fish [12].

### Study period and location

This study was conducted from May 2021 to October 2021. The experimental study was conducted in Animal Laboratory, Department of Biology, Faculty of Science and Technology, Universitas Airlangga.

### Experimental design

Adult tilapia (*Oreochromis niloticus*) weighing between 200 and 250 g obtained from the Technical Implementation Unit for the Development of Umbulan Freshwater Aquaculture, Pasuruan, East Java, Indonesia, were fed commercial pellets (30% protein, 3% fat, and 4% fiber, Takari, Sidoarjo, Indonesia). They were acclimatized for 2 weeks in the laboratory before treatment. Thirty-six tilapia fishes were divided into 12 groups, including one control group (fed with commercial pellets only), three negative control groups (exposed to various concentrations of PP), four treatment groups (fed with a mixture of commercial-probiotic pellets [200 mL/kg, 1×10^8^ colony-forming unit (CFU)/mL] and exposed to PP of various concentrations), and four treatment groups (fed with commercial vitamin C pellets [100 mg/kg] and exposed to PP of various concentrations). Each group consisted of three fishes. The variation of PP (<74 μm) concentration was selected based on the results of a previous study by Soares *et al*. [13] The PP concentrations used in the study were 0, 0.1, 1, and 10 mg/L and the treatment time was 2 weeks. Fishes were fed twice a day, morning and evening, for 2 weeks.

### Steroid analysis

After 2 weeks of treatment, blood samples were collected for analysis on the 15^th^ day. Fish blood was collected through a caudal vein or artery using a syringe. The whole blood was collected in a 1.5 mL microcentrifuge tube (Eppendorf) and centrifuged at 1006×g and 4°C for 10 min. The upper layer containing serum was collected. Testosterone and estradiol levels in the serum were measured using the sandwich - enzyme-linked immunosorbent assay method (Bioassay Technology Laboratory, Shanghai, China), according to the manufacturer’s protocols. The absorbance was measured using a microplate reader at 450 nm. Finally, the testosterone and estradiol levels were determined using a standard curve.

### Histology of gonad processing

Tissue gonads (testes) were harvested on the 15^th^ day for histopathological analysis. All testis samples from each fish were collected and fixed in a 10% neutral buffered formalin solution. After 24 h of fixation, testis tissues were prepared for serial sectioning. All the tissues were processed using the standard operating procedure for histology [14]. Tissue sections of approximately 4 mm thickness from each tissue were cut with a semi-automatic microtome, embedded in paraffin. They were mounted on Dibutylphthalate Polystyrene Xylene, stained with hematoxylin and eosin, and analyzed under a light microscope (Olympus, Japan) [15]. Testicular histopathological alterations were assessed qualitatively by comparing the control group with various concentrations of PP and supplements. In addition, the diameter and circumference were measured using a digital camera microscope (Optilab, Miconos, Indonesia). The diameter of the seminiferous tubules, the diameter around the cyst, the diameter of each cyst containing spermatid cells, and the number of spermatogenic cells (spermatogonia, spermatocytes, and spermatids) in the cyst were individually measured.

### Statistical analysis

Statistical analysis was performed using a two-way analysis of variance (each test being conducted at 0.05% level of probability) using the Windows Statistical Package for the Social Sciences software v.13 (IBM Corp., New York, USA).

## Results

The measured estradiol and testosterone levels indicated a different effect on fish treated with various PP concentrations and supplementary feed. No significant difference was observed in fishes exposed to PP concentrations of up to 1 mg/L; however, estradiol levels decreased at higher concentrations (10 mg/L) of PP (Table-1). Moreover, the result also shows that feeding with probiotic supplements and vitamin C can restore estradiol levels. Under normal conditions (without PP), probiotic supplementation is reportedly better at increasing estradiol levels than vitamin C. This is in contrast to the observations following exposure to PP of different concentrations. Meanwhile, in general, our study results showed that giving vitamin C supplements to fishes exposed to various PP concentrations was better than giving probiotics, except for exposure to concentrations of 0.1 and 1 mg/L.

**Table 1:** Tilapia fish steroid levels in various concentrations of PP and feed supplements

Steroid (ng/L)	Polystyrene particles (mg/L)	Commercial feed	Commercial feed-probiotics	Commercial feed-vitamin C
Estradiol	0	11,07±0,10^b^	14,85±1,76^d^	13,36±0,10^c^
	0.1	11,88±1,53^b^	18,00±2,20^d^	17,00±0,86^d^
	1	9,85±1,91^b^	12,00±2,70^c^	12,82±2,10^c^
	10	7,15±0,10^a^	14,18±0,10^c^	24,00±0,10^e^
Testosterone	0	452,15±10,00^c^	498,00±25,00^d^	487,00±10,00^d^
	0.1	434,00±11,00^c^	478,00±20,00^d^	481,36±13,00^d^
	1	374,76±10,00^b^	327,00±50,00^b^	405,42±5,00^c^
	10	341,00±8,00^a^	402,00±20,00^c^	446,00±10,00^c^

Different letters (a, b, c, and d) show significant difference

Testosterone levels in the group exposed to a PP concentration of 0.1 mg/L remained unaltered, compared with the control group. However, the testosterone level decreased significantly when the PP concentration was increased to 1 mg/L or more. Hence, it can be asserted that probiotic and vitamin C supplements could increase testosterone levels under normal conditions (control). This observation was also made when the fishes were exposed to PP concentrations of 0.1 mg/L and 10 mg/L.

**Figure-1:**
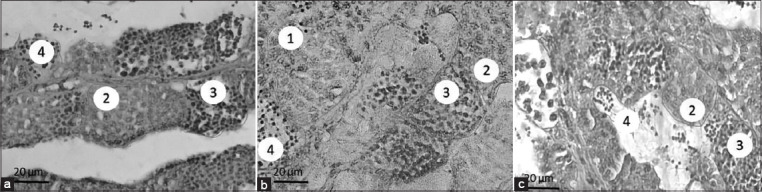
Photomicrograph of seminiferous tubules of fish testes in control groups. a. Commercial feed only; b. Mix commercial feed and probiotics; c. Mix commercial feed and vitamin C. 1. Spermatogonium; 2. Spermatocyte; 3. Spermatid; 4. Spermatozoa. Hematoxylin and eosin stain

**Figure-2:**
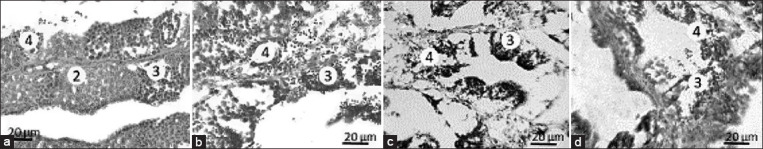
Photomicrograph of seminiferous tubules of fish testes after exposure with various concentrations of polystyrene particles (PP) and feeding with commercial feed only. a. control; b. 0.1 mg/L; c. 1 mg/L; d. 10 mg/L PP. 2. Spermatocytes; 3. Spermatids; 4. Spermatozoa. Hematoxylin and eosin stains.

**Figure-3:**
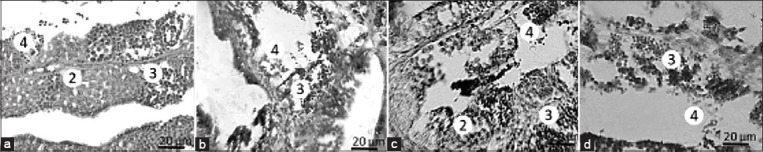
Photomicrograph of seminiferous tubules of fish testes after exposure to 10 mg/L of polystyrene particles and treated with a various mix of supplemental feed. (a). Seminiferous tubules in control group, (b) seminiferous tubules with commercial feed only, (c) commercial feeds with probiotics, (d) commercial feed with vitamin C (2. Spermatocytes; 3. Spermatids; 4. Spermatozoa in Hematoxylin and eosin staining).

The histopathological analysis of fish gonads showed no change in the structure of the cysts and seminiferous tubules in the control group (variation of feed supplements without PP exposure). The structure of the seminiferous tubules was good and consisted of many cysts. Inside the cysts, there is a variation in the development of spermatogenic cells, with spermatogonia appearing to be the largest compared with the other spermatogenic cells (Figure-1b-1). Cysts consisting of spermatocytes also appear to exhibit a characteristic round shape and condensed nucleus (Figure-1a-2). Spermatids and spermatozoa within the cysts appear smaller than other spermatogenic cells (Figure-1a-3; 3b-3; 3c-3). Spermatozoa, previously found in the cysts, were also discovered in the lumen of the seminiferous tubules (Figure-1c-4).

The effect of PP exposure on the testicular structure is a change in the cyst structure in the seminiferous tubules. Compared with the control (Figure-2a), the cyst structure of the group exposed to PP of various concentrations appeared to have a change in shape and a decrease in size. In addition, the spermatogenic cells inside were not compact (Figure-2b-d). Exposure to PP can reduce the permeability of the cyst membrane, which leads to membrane damage or cyst rupturing.

Exposure to high concentrations of PP (10 mg/L) increased the damage of cysts (Figure-3b-d), compared with the control (Figure-3a). However, there was a significant decrease in cyst damage for the group supplemented with probiotic feeds (Figure-3c), compared with the group without supplementary feed (Figure-3b). A similar result was also observed for the group supplemented with vitamin C feed (Figure-3d).

Measurements of the cells, tissues, and organs that make up the testes are presented in Table-2. Tilapia fish testes are composed of seminiferous tubules extending from the testicular basement membrane to the testicular lumen. The diameter of the seminiferous tubules showed no significant difference (p>0.05), compared to the control group and the group exposed to a PP concentration of 0.1 mg/L fed with commercial feeding only. However, a higher concentration of PP reduces the tubular diameter significantly (p<0.05). The group treated with probiotic supplement feed (a mixture of commercial feed and probiotics) showed an increase in the diameter of the seminiferous tubules of the fish. A similar result was also observed for the group treated with vitamin C supplements (a mixture of commercial feed and vitamin C).

**Table 2:** The size of seminiferous tubules, cysts, and spermatogenic cells in the testicles of fish treated with various PP concentrations and feeding types.

Sizes (µm) and a total of cells	Polystyrene particles concentration (mg/L)	Commercial feed	Mix commercial feed and probiotics (200 mL/kg feed)	Mix commercial feed and vitamin C (100 mg/kg feed)
**Diameters**				
Seminiferous tubules	0	1801.58±34.64^b^	1919.26±10.35^cd^	2033.10±11.16^d^
	0.1	1752.22±34.50^b^	1859.89±10.36^c^	1882.63±19.34^c^
	1	1466.62±159.57^a^	1434.64±167.19^b^	1896.13±18.63^c^
	10	1449.94±171.83^a^	1738.45±142.39^b^	1844.48±16.76^c^
Cyst in each seminiferous tubules	0	363.13±70.70^b^	466.35±73.02^d^	430.02±33.52^c^
	0.1	351.96±48.66^b^	436.57±59.16^c^	423.39±31.53^c^
	1	392.01±22.28^b^	423.20±63.44^c^	427.14±61.30^c^
	10	333.05±18.21^a^	356.92±33.44^b^	369.40±26.72^b^
Around of the cyst in each seminiferous tubules	0	1312.52±50.00^a^	1489.77±80.00^b^	1480.64±25.00^b^
	0.1	1284.78±120.00^a^	1511.07±40.00^b^	1516.01±11.98^b^
	1	1391.10±98.96^a^	1497.73±80.00^b^	1394.01±70.00^a^
	10	1267.57±50.00^a^	1258.75±30.00^a^	1243.73±80.00^a^
Thickness of seminiferous tubular epithelial	0	432.07±20.26^c^	471.56±55.25^c^	527.77±38.99^d^
	0.1	376.36±10.53^b^	451.83±30.49^c^	513.07±27.45^d^
	1	360.31±15.27^b^	433.48±24.02^c^	451.74±33.11^c^
	10	341.95±12.62^a^	425.82±72.99^c^	440.41±21.58^c^
**Cells Diameter**				
Spermatogonia	0	50.01±2.95^b^	52.13±0.69^b^	59.23±4.41^c^
	0.1	50.27±2.68^b^	52.04±0.75^b^	59.90±4.83^c^
	1	49.79±0.71^a^	49.30±2.88^a^	53.70±0.10^b^
	10	46.10±4.77^a^	47.97±2.25^a^	50.54±0.19^a^
Spermatocytes	0	44.75±1.85^b^	48.57±3.27^c^	56.76±1.65^d^
	0.1	43.50±1.7^b^	50.25±2.48^c^	54.32±1.97^d^
	1	32.62±1.78^a^	36.42±2.17^a^	36.98±1.56^a^
	10	37.02±4.94^a^	40.76±1.73^a^	32.30±7.32^a^
Spermatids	0	27.16±1.57^c^	25.49±0.42^c^	29.09±4.75^c^
	0.1	22.56±0.97^b^	25.99±2.36^c^	27.93±6.80^c^
	1	20.79±1.05^a^	29.44±3.80^c^	40.00±8.30^c^
	10	20.55±1.06^a^	24.64±0.22^b^	33.68±7.71^c^
**Number of cells per cyst**				
Spermatogonia	0	8.25±2.87^b^	11.33±1.53^b^	24.67±1.15^d^
	0.1	8.00±1.65^b^	8.67±3.79^b^	22.00±4.36^d^
	1	6.33±1.53^b^	7.67±1.53^b^	15.33±0.58^c^
	10	2.33±0.58^a^	5.33±0.58^b^	9.00±1.00^b^
Spermatocytes	0	26.00±2.00^b^	29.00±1.00^c^	36.25±1.71^d^
	0.1	17.67±0.58^a^	22.67±2.31^b^	34.75±0.96^d^
	1	15.00±4.36^a^	19.33±1.53^a^	27.00±1.36^b^
	10	13.75±2.87^a^	17.33±0.58^a^	23.33±0.58^b^
Spermatids	0	41.70±3.85^b^	45.33±4.43^c^	72.00±7.81^d^
	0.1	31.00±3.08^b^	45.00±1.00^c^	71.00±7.55^d^
	1	29.33±1.15^b^	37.67±7.00^b^	63.00±5.29^d^
	10	23.33±3.07^a^	38.33±7.02^b^	43.00±1.00^c^

Different letters (a, b, c, and d) show a significant difference

In the group treated with a PP concentration of 10 mg/L, the diameter of the tilapia cyst was decreased significantly. Treatment with both probiotics and vitamin C supplements feed improved the diameter of the cysts. The cyst circumference result showed no significant change after exposure to various PP concentrations. However, treatment with supplementary feed either improves or increases the size of cyst circumference. This observation is in contrast to the thickness of the seminiferous tubular epithelium, which decreased after exposure to various PP concentrations. The higher the concentration of PP, the lower the thickness of the seminiferous tubular epithelium.

The diameter of spermatogenic cells inside the cyst varies for each cell type. Spermatogonia have the largest diameter, followed by spermatocytes, spermatids, and spermatozoa. PP exposure affects the diameter of spermatogenic cells. However, treatment with a probiotic supplement or vitamin C can restore the diameter of the spermatogenic cells. A similar result was observed for the number of spermatogenic cells in each cyst. The higher the PP concentration, the lower the number of spermatogenic cells. This includes the spermatogonia and spermatocytes, although the number of spermatids in each cyst remained constant. Supplementary feeding restored the number of spermatogenic cells in the cyst after exposure to plastic particles.

## Discussion

Considering PP activities as carriers of environmental pollutants, studies using fish have revealed that PP (<74 mm) affected estradiol and testosterone levels, which regulated the development of male tilapia reproductive organs (Table-1). PP are toxic substances affecting gene expression, especially the estradiol receptor alpha (ERα). Thus, exposure to native plastic particles reduced ERα mRNA levels compared with controls [16]. A decrease in the level of ERa reduces the activity of glandular cells to produce steroid hormone (estradiol).

PP also affect the cell and tissue structure of fish testes (Figures-1-3). In addition, there is also a change in the size of the seminiferous tubules and cysts that constitute the testes. Moreover, PP also affect the size of the spermatogenic cells in the cyst (Table-2). The process of spermatogenesis of tilapia fishes takes approximately 10-11 days at a temperature of 25°C; spermatocyte development occurs within 5 days, and spermiogenesis occurs between 5 and 6 days. In this study, the PP exposure carried out for 14 days observably affected spermatogenic development. The spermatogonia (2n) were suspected to have a metabolic disorder that could inhibit the mitotic process (2n). Thus, the number of spermatocytes was lower in the test fish compared with the control. The presence of disturbances and a decrease in the function and number of spermatocytes can interfere with the meiosis process: thus, the number of spermatids also decreased following PP exposure.

The physical effects of PP accumulation are more complex than the toxicological effects of poison when it enters the body. Plastic particles (e.g., polyvinyl chloride [PVC]) decrease the enzymatic activity of gut microbes [17]. The smaller PP consumed by fish is opined to be absorbed by the intestinal epithelium and other food extracts. This is because PP can bind to organic and inorganic molecules. Hence, PP can also carry other pollutants, including hydrophobic organic pollutants and heavy metals [6].

According to Bandow *et al*. [4], oxidized PP can dissolve in water. This change is related to the oxidation process accompanied by the formation of a new adsorption band (C=O, C–O, and OH). Meanwhile, as mentioned in Wang *et al*. [18], particles have toxic properties depending on their size. Thus, these toxic particles increase the inflammatory response and bioaccumulation and transport organic pollutants into the body. The shape and function of the surface, size, surface charge, and hydrophobic properties of the particles can predict the uptake of PP [19]. PP possesses certain characteristics that facilitate movement across living cells, such as dendritic cells, into the lymphatic or circulatory system. On entering a system, they accumulate in secondary organs, affecting the body cells and immune system [20,21].

PP enters gastrointestinal epithelial cells through endocytic and persorption pathways [22]. Wright and Kelly opined that ingested PP can cause tissue inflammation, cell proliferation, and necrosis and result in weakened immune cells. Furthermore, according to Panti *et al*. [16], PP show a toxicological impact on the liver or changes in intestinal tissue. These findings were supported by polymerase chain reaction on fish exposed to mean platelet volume (MPV) and PVC, and milk protein isolate. The expression of tumor necrosis factor receptor-associated factor 3 (TRAF3) and peroxisome proliferator activator receptor (PPARα/γ) gene showed that TRAF3 expression decreased with increasing exposure time. TRAF3 expression upregulated the genes associated with PP-contaminated food pellets. In contrast, PPARa gene expression increased with a higher exposure time. PPARg appears to be the most affected following MPV exposure, thus showing an effect due to the leaching of plastic additives from PVC.

According to Deng *et al*. [23], as a carrier, PP transports phthalate ester contaminants into the organism, where they build up in the intestine, thus causing serious health risks. Furthermore, most of the PP contains a chemical additive called bisphenol-A (BPA) [24], which causes adverse effects on the health of aquatic organisms by altering endocrine functions. That is, they exert estradiol and genotoxic effects. Based on its widespread occurrence, BPA poses a high risk to humans exposed to it through the consumption of aquatic organisms exposed to PP (e.g., fish and shrimp). In addition, to disrupt intestine function, PP also causes oxidative stress and impaired lipid metabolism in the liver [25].

This study indicated that the administration of probiotic and vitamin C supplements can restore estradiol and testosterone levels in fishes exposed to PP. Probiotic supplements containing lactic acid bacteria can absorb PP that bind to organic compounds in the digestive tract, which will further be excreted out of the body along with fish feces. Lactic acid bacteria can enzymatically neutralize toxic materials in their cells, including PP, which can be degraded into non-toxic materials. As mentioned in Gong *et al*. [26], changes in PP after biodegradation indicate a layer-by-layer bio-decomposition from outer to inner particle layers. Probiotic bacteria can dispel and degrade PP and BPA into non-toxic compounds. Probiotics can grow on media containing PP, and therefore, these bacteria can be added to the feed given to the cultured organism. In addition to degrading PP, supplementing with probiotic bacteria will provide enhanced benefits such as increased growth, development, and resistance to disease of the cultivated host.

In this study, adding vitamin C as a dietary supplement could restore the size of the seminiferous tubules, cysts, and spermatogenic cells. vitamin C also increases the number of spermatogenic cells (Table-2). vitamin C is a natural organic compound with antioxidant properties [27]. It functions as a redox buffer that can reduce and neutralize reactive oxygen species generated by PP. It is a cofactor for enzymes involved in regulating hormone biosynthesis and regeneration of other antioxidants involved in signal transduction. These molecules play crucial roles in several physiological processes, such as immune stimulation, neurotransmitters, and iron absorption. vitamin C also plays a role in detoxifying heavy metals in the body.

## Conclusion

Our study results indicated that PP exposure (<74 mm) could reduce estradiol (10 mg/L, PP) and testosterone (1 mg/L, PP) levels and thus change the structure, shape, and size of the cells and cysts of tilapia testes. This effect of PP on fishes can be ameliorated by feeding them with supplements containing probiotics (200 mL/kg) and vitamin C (100 mg/kg). These findings are crucial considering the heavy presence of PP in polluted waters that enter and accumulate in the fish body. On accumulation in the body, they can interfere with the reproductive health of the fish and, in turn, impact human health due to exposure through consumption of the fish. Hence, we strongly recommend using probiotics and vitamin C as feed supplements for fish farming in water bodies contaminated with toxic materials.

## Authors’ Contributions

AH and MP: Participated in conceptualization, experimental design, and drafting of the manuscript. ARA, MRK, SM, and WA: Experimentation and data collection. HS and FRPD: Data analysis. AH and MP: Preparation and revision of the manuscript. All authors read and approved the final manuscript.
